# Bilateral, Neglected Lateral Humeral Condyle Nonunion: A Unique Case of Cubitus Valgus and Ulnar Neuropathy Successfully Treated With Combined Closed Wedge Osteotomy and Ulnar Nerve Transposition

**DOI:** 10.7759/cureus.77870

**Published:** 2025-01-23

**Authors:** Ahmet Burak Satılmış, Tolgahan Cengiz, Ahmet Ülker, Zafer Uzunay, Ercan Bayar

**Affiliations:** 1 Orthopaedics and Traumatology, Taşköprü State Hospital, Kastamonu, TUR; 2 Orthopaedics and Traumatology, Mersin University, Mersin, TUR; 3 Orthopaedics and Traumatology, Medicalpark Adana Hospital, Adana, TUR; 4 Orthopaedics and Traumatology, Tosya State Hospital, Kastamonu, TUR

**Keywords:** anterior transposition of the ulnar nerve, closing wedge osteotomy, cubitus valgus deformity, lateral humeral condyle fracture, ulnar nerve neuropathy

## Abstract

Lateral humeral condyle (LHC) fractures are frequent elbow injuries in children, and if neglected or inadequately managed, they can lead to complications such as cubitus valgus deformity, ulnar nerve issues, and instability of the elbow joint. This case report presents a 20-year-old male with bilateral neglected LHC nonunion, leading to severe cubitus valgus deformity (45° on the left, 41° on the right) and ulnar neuropathy. The patient underwent a combination of 35° closed wedge osteotomy and anterior transposition of the ulnar nerve (ATUN) using a posterior approach. Fixation was achieved with locking plates. Postoperatively, significant improvement in ulnar nerve function, full elbow motion, and satisfactory cosmetic results were observed. Complete osteotomy healing occurred at nine months, with preparations for contralateral surgery underway. The presence of fractures in both elbows with identical deformities makes this case unique in the literature. This case highlights the efficacy of combining closed wedge osteotomy with ATUN in a single procedure, offering a practical, less complex solution for neglected LHC nonunion with favorable outcomes in deformity correction and nerve function restoration.

## Introduction

Lateral humeral condyle (LHC) fractures are common pediatric elbow injuries, yet the management of neglected or untreated fractures remains challenging and controversial. Nonunion of LHC fractures can lead to significant complications such as cubitus valgus deformity, ulnar nerve palsy, and limited elbow function [1-4]. These complications affect quality of life and complicate surgical intervention due to risks like decreased range of motion and avascular necrosis of the lateral humeral condyle [5]. In cases of severe cubitus valgus deformity with ulnar nerve paralysis, combining anterior transposition of the ulnar nerve (ATUN) with corrective osteotomy has been reported as an effective treatment option [6-8]. This approach addresses both the deformity and its neurological complications. In this rare case report, we present a unique adult patient with bilateral cubitus valgus deformity and ulnar nerve paralysis due to neglected LHC fractures in childhood. The patient was successfully treated with ATUN combined with corrective closing wedge osteotomy. This report highlights the challenges and outcomes of managing complex, bilateral neglected LHC fractured with associated complications.

## Case presentation

**Figure 1:**
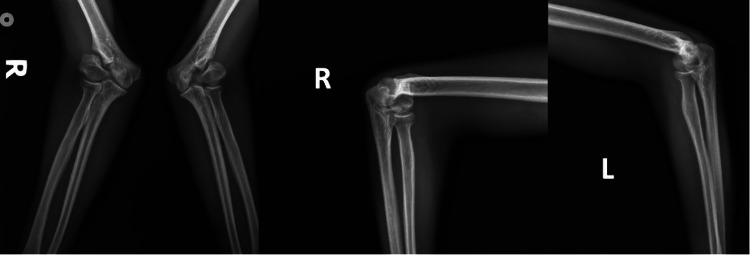
Radiographic evaluation revealed non-union in both elbows caused by previous LHC fractures, accompanied by pathological valgus deformities LHC: lateral humeral condyle

A 20-year-old male patient applied to the outpatient clinic with complaints of progressive deformity in both elbows, numbness in the fingers, and loss of sensation. The patient's history revealed that he fractured his left elbow when he was three years old and his right elbow when he was four years old and was treated with a cast without surgery. He had no chronic disease in his medical history and stated that he smoked a pack of cigarettes a day. In the physical examination, both elbows had full flexion but there was a 10-degree extension loss and cubitus valgus deformity along with sensory loss in the UN region, marked intrinsic muscle atrophy, claw deformity, and positive Froment signs. Radiographic examination revealed non-union in both elbows due to previous LHC fractures, a 45-degree pathological valgus angle in the left elbow, and a 41-degree pathological valgus angle in the right elbow (Figure 1). UN was tested with electromyography (EMG) and detected delayed sensory latency.

**Figure 2:**
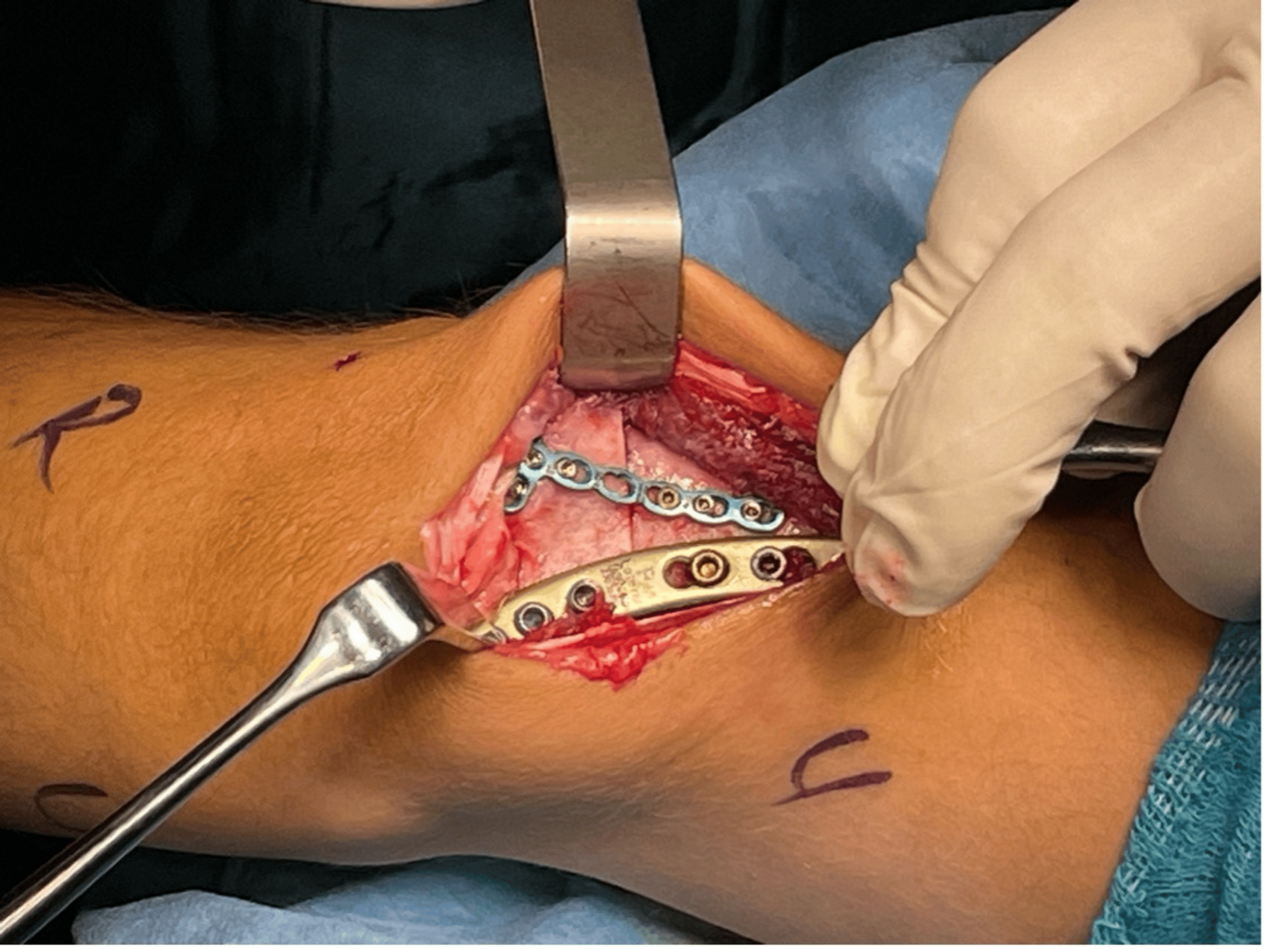
Image taken during surgery

**Figure 3:**
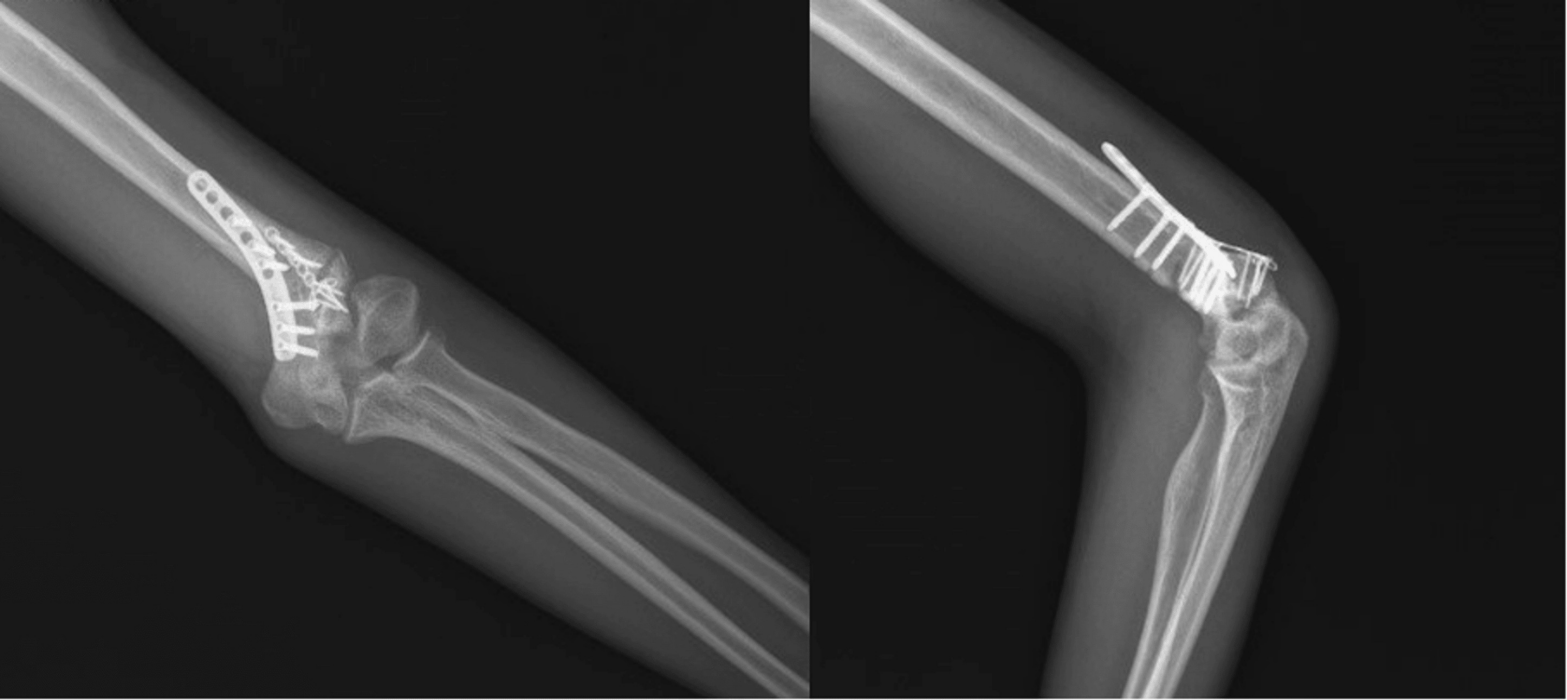
Radiographic images of the patient taken on the first postoperative day

**Figure 4:**
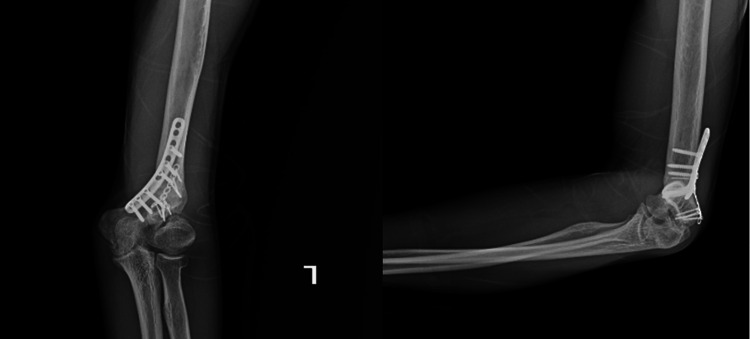
Postoperative radiographic images of the patient taken at nine months

**Figure 5:**
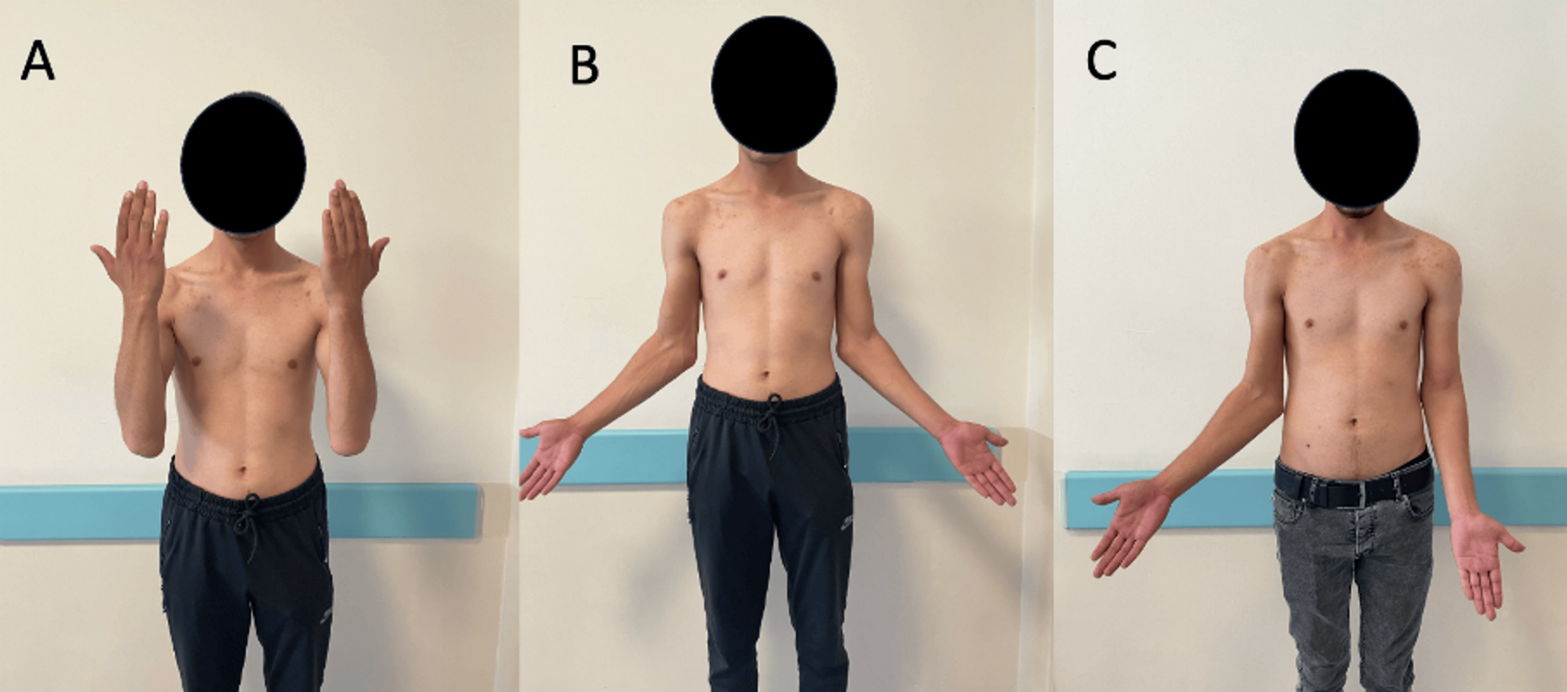
Preoperative (A-B) and postoperative (C) photographs of the patient's elbow

Surgery was planned for the patient's left elbow cubitus valgus deformity and ulnar nerve paralysis. As a result of preoperative planning, we found removing a 35-degree, 2-cm high wedge targeting a valgus angle of 10 degrees was appropriate. Then, a posterior approach was used to reach the elbow joint to treat the deformity and UN paralysis. It was observed that the UN was attached to the surrounding soft tissues, but there was no obvious nerve abnormality. Since traction-type neuropathy was considered, the UN was transposed anteriorly subcutaneously. Then, a 35-degree corrective closed wedge osteotomy was applied to the distal humerus, and fixation was achieved using two locking plates (Figure 2). LHC non-union was not intervened to disrupt the joint harmony developed during the patient's developmental period. The elbow was found stable due to fixation with the plate, and he was put in a bandage without applying a cast. Immediately after the surgery, the patient's sensory deficit and numbness in the UN trace were improved. On the radiograph taken on the first postoperative day (Figure 3), the left elbow valgus angle was measured at 10 degrees as planned. The patient was closely followed up, and at the end of nine months, complete union was achieved at the osteotomy line (Figure 4). After union, a satisfactory elbow appearance regained grip strength, full elbow range of motion (0-150°), and complete recovery of sensory functions of the ulnar nerve was achieved (Figure 5). The patient's follow-up for his left elbow has just ended, and preparations for surgery for his right elbow have begun.

## Discussion

Neglected nonunion of pediatric LHC fractures can lead to progressive cubitus valgus deformity of the elbow and the development of late ulnar nerve neuropathy with pain and instability of the elbow [5,9]. Nonunion of LHC fractures has been reported to be the cause of late ulnar nerve palsy in 25% of cases of ulnar neuritis [10]. The management of LHC nonunion remains controversial due to the high reported complication rates [5,7]. Various treatment options have been used to manage cubitus valgus associated with nonunion of LHC. For example, a case series published by Abed et al. described the combined triple management (fixation of the nonunion site, dome corrective osteotomy, and anterior transposition of the ulnar nerve) for neglected LHC non-union [8]. In this case report, 17 years have passed since the patient's LHC fracture, and the pseudoarthrosis of the humeral condyle was not treated because it was not symptomatic. Although some authors recommend osteosynthesis of pseudoarthrosis, good results have been reported in the literature without surgical treatment [11].

The three most commonly published osteotomy methods for cubitus valgus are closed wedge osteotomy, V-shaped translational osteotomy, and dome osteotomy. Translational osteotomy has the advantage of providing natural stability due to V-shaped cuts and multiplanar correction [12]. Kin et al. reported the results of 13 patients with this technique, which achieved good angular correction and improved medial protrusion with a final valgus angle of 9.1°. However, four patients required an additional osteotomy to correct flexion contracture. Dome osteotomy is less complex than the previous technique but is less sensitive in correction than the wedge removal method [13]. Hahn et al. corrected the valgus angle from 24° to 11° in 13 patients with this osteotomy, improving clinical-functional scores and medial protrusion [14]. Monoplanar wedge osteotomy is frequently criticized in the literature because the deformity usually has a multiplanar structure, and therefore, many authors argue that the correction should also be multiplanar. Closing wedge osteotomy is monoplane and does not allow correction in all planes [15,16]. Criticisms of this osteotomy include that it does not provide rotational correction, offers less stability because it is a linear osteotomy, and increases the medial protrusion because it does not allow displacement of the distal fragment.

Although translational and dome osteotomy types offer the advantage of multiplanar correction, there are no comparative studies with monoplane osteotomy. Therefore, there needs to be a clear consensus in the literature. We preferred lateral closing wedge osteotomy because it is a less complicated technique than multiplanar osteotomies, and patient satisfaction is high. We think that monoplane correction offers similar results to multiplanar techniques and is a more straightforward method, except in exceptional cases with significant deformities outside the coronal plane. Unlike the double medial and lateral incisions used in traditional LHC non-union techniques, we used a single posterior midline skin incision to address all problem components. The paratricipital approach provides easy access to both sides of the elbow without disrupting the elbow extensor mechanism [17]. The nonunion region can be adequately exposed through the lateral side of the approach. The osteotomy line can be created through the approach's medial and lateral sides. The ulnar nerve can be transposed through the medial part of the approach. Tien et al. also used a midline triceps approach to treat cases of cubitus valgus associated with LHC nonunion [18].

Ulnar nerve pathologies are common in cubitus valgus deformity. Anterior transposition of the ulnar nerve has been used alone to relieve ulnar nerve neuropathy but did not relieve either elbow pain or instability [7]. Supracondylar corrective osteotomy of the distal part of the humerus has been used alone to relieve tension on the ulnar nerve but did not relieve either elbow pain or instability and did not prevent the progression of the valgus deformity [7,18]. In this case report, we corrected the deformity and transposed the ulnar nerve in the same session, thus achieving complete correction of ulnar nerve paralysis.

## Conclusions

In conclusion, neglected nonunion cases of pediatric lateral humeral condyle fractures may lead to serious complications such as cubitus valgus deformity and ulnar nerve neuropathy. The surgical approach should be carefully planned in managing such cases, and neurological symptoms and deformities should be addressed simultaneously. With the combined method used in our study, the deformity was successfully corrected, and ulnar nerve functions were wholly improved thanks to the closing wedge osteotomy and anterior transposition of the ulnar nerve. Despite the frequently criticized limitations in monoplane osteotomies, patient satisfaction was ensured, and satisfactory functional results were obtained with good surgical planning and technical application. This method can be considered a practical option in suitable cases because it is less complicated than multiplanar techniques and provides high patient satisfaction.
